# Supermacroporous Composite Cryogels in Biomedical Applications

**DOI:** 10.3390/gels5020020

**Published:** 2019-04-17

**Authors:** Yeşeren Saylan, Adil Denizli

**Affiliations:** Department of Chemistry, Hacettepe University, 06800 Ankara, Turkey; yeseren@hacettepe.edu.tr

**Keywords:** biomedical application, composite, cryogel, polymer, supermacroporous

## Abstract

Supermacroporous gels, called cryogels, are unique scaffolds that can be prepared by polymerization of monomer solution under sub-zero temperatures. They are widely used in many applications and have significant potential biomaterials, especially for biomedical applications due to their inherent interconnected supermacroporous structures and easy formation of composite polymers in comparison to other porous polymer synthesis techniques. This review highlights the fundamentals of supermacroporous cryogels and composite cryogels, and then comprehensively summarizes recent studies in preparation, functionalization, and utilization with mechanical, biological and physicochemical features, according to the biomedical applications. Furthermore, conclusions and outlooks are discussed for the use of these promising and durable supermacroporous composite cryogels.

## 1. Introduction

A biomolecule presents in organisms, essential to some typically biochemical processes, such as cell division, morphogenesis, and development. The evolution of new approaches for separation, recognition, and purification of biomolecules has been necessary for numerous of the latest advancements in biomedical applications. A broad diversity of purification, separation and recognition techniques are presented today [1]. The biomolecules can be purified from crude extracts by several methods, including affinity chromatography, liquid chromatography, gas chromatography, gel-filtration chromatography, and ion-exchange chromatography [2]. Efficient and inexpensive techniques for separation, recognition, and purification of target biomolecules are necessary to provide purely for biomedical applications. Traditional techniques which exploit differences in size, shape, and density have low specificity and are difficult to scale up [3]. 

Cryotropic gelation is a process of gel formation and has been used in order to produce supermacroporous hydrophilic gels called cryogels. Cryogels are interconnected supermacroporous materials that can be synthesized at sub-zero temperatures by using an available initiator/activator pair [4,5]. The polymerization is carried out in frozen monomer solution, where it occurs in interstitial places between the ice crystals. The crystals serve as a porogen throughout the scaffold during polymerization and a supermacroporous polymer is formed after the thawing process [6,7]. The significant properties of cryogels, such as large pores (10–200 μm), short diffusion paths, flexibility, good biocompatibility, and high mechanical strength, that make them efficient carriers for immobilization of biomolecules [8,9]. Cryogels typically have interconnected supermacroporous polymers that permit unhindered diffusion of solutes, as well as mass transport of particles. Composite cryogels can be prepared by combining with other polymeric materials and these polymers can be utilized in various biomedical applications, including drug delivery [10], tissue engineering [11], protein purification [12], enzyme activity [13]. Thus, the supermacroporous composite cryogels are specifically designed for application in technologically challenging bioseparation processes and high-throughput analysis [14]. In this review, the supermacroporous cryogels and composite cryogels are described and then some specific studies are discussed according to their applications and contributions to literature.

## 2. Supermacroporous Cryogels and Composite Cryogels

The first mention of flowing reactions in frozen systems belong to the 1930s, but a larger contribution to this subject began only in the 1960s [15]. A classical concept of frozen solutions as polycrystalline solids at temperatures below the eutectic degree of temperature does not explain the flow of a different mechanism of chemical reactions in such systems. The first who suggested that freezing solutions in the systems along with a solid phase leaves liquid regions between the crystals of solvent, were Butler and Bruce [16]. Later in the study, kinetics of the reaction of low molecular weight substances in crystallized solvents will be discussed. Pinkok Kiovski also concluded that these reactions take place in the liquid inclusions containing high concentrations of reagents, located between the crystalline solids [17,18]. 

**Figure 1:**
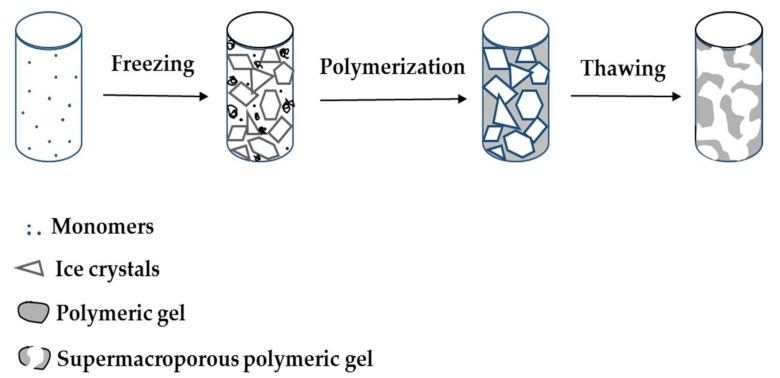
Illustration of supermacroporous cryogel preparation. Republished with permission from [23]. Copyright 2004 Elsevier.

**Figure 2:**
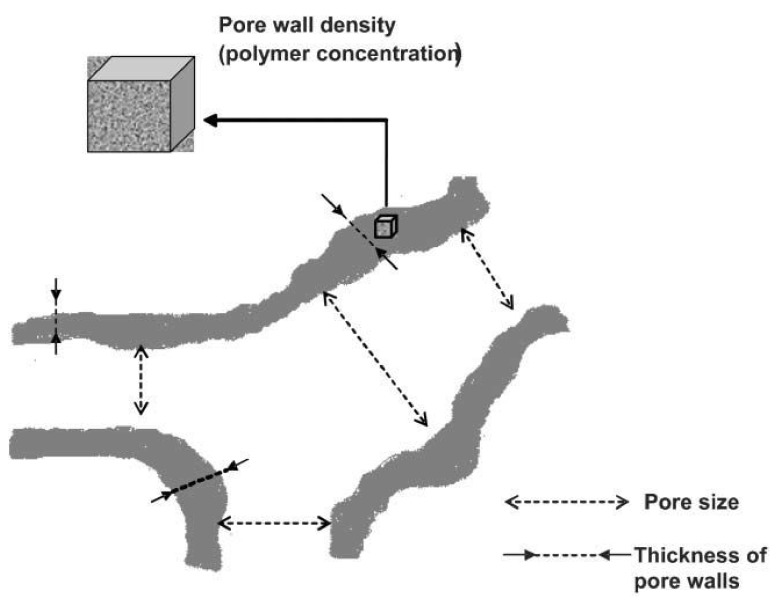
Scheme of thickness and density of supermacroporous cryogel pore size effects. Republished with permission from [19]. Copyright 2005 the Royal Society of Chemistry.

As depicted in Figure 1, the cryogel preparation includes the following steps: Freezing a polymeric solution including cross-linker and initiator, holding its state frozen for pore formation, cross-linking of compounds forming macropores and thawing at room temperature. Aqueous media is frozen, and the polymerization occurs in a non-frozen liquid microphase. Next, ice crystals melt at room temperature and form large, continuously interconnected pores. These interconnected macropores allow the flow of mobile phase with minimal flow resistance (Figure 2). They have several advantages for purification, separation, and recognition of biomolecules. Pore size of the cryogel is mostly affected by the initial concentration of monomers, their physicochemical properties, freezing degree, and rate. The pore size of cryogels ranges from a few to tens of hundreds micrometers [19,20]. The range of pores from 10 to 200 μm is referred to as supermacropores in the literature [21]. Up to 90% of the swollen cryogel volume is filled with water. It means that 90% of the weight is the water located throughout the pores of the scaffold. Hydrated polymer (pore walls) constitutes only 10% of the total volume. A high polymer concentration in pore walls endorses them with mechanic stability making whole cryogel elastic and spongelike [22].

Cryogels give better results for separation of proteins, nucleic acids and polysaccharides from their natural sources when compared with conventional polymeric beads. Different ligands can be immobilized on cryogels for separation, purification or removal of several substances [24]. In the last decade, cryogels were prepared in different forms and many ligands, such as concanavalin A, protein A, tryptophan, histidine, triazine dyes, and transition metal ions, were immobilized on the cryogels. These ligand immobilized cryogels were used for separation of many macromolecules, such as immunoglobulin G, lysozyme, albumin, DNA, cytochrome c, cholesterol, and lectins [25,26,27,28,29,30,31,32]. In addition, they are attractive supports for cell immobilization because of their physicochemical properties and high porosity [33]. Polyacrylamide and poly(vinyl alcohol) cryogels are used for cell entrapment or adsorption. They have a suitable three-dimensional structure for cell growth and proliferation. Surface properties of support materials used especially in tissue engineering have a great effect on cell affinity. As cryogels are hydrophilic and porous, they are very attractive materials for cell affinity [34].

Cryogels with large specific surface areas for biomolecule purification are expected to possess high binding capacity. Preparation of a composite cryogel with embedded particles offers an attractive arrangement because good accessibility is offered and low back-pressure is maintained. The composite cryogels offer large pores with convective flow and thus, good mass transfer conditions and represent affinity binders. Thus, composite cryogels are the most eligible candidates for extraction due to their high compatibility with aqueous as well as biological systems. They are also resistant to a broad range of buffer systems which ultimately adds to their reliability. Moreover, composite cryogels possess high toughness and superfast response that make the extraction process robust and quick [35,36,37].

## 3. Composite Cryogels as Versatile Tools for Biomedical Applications

Numerous supermacroporous composite cryogels have been developed in recent years. These structures depend on natural and synthetic polymers employing various types of synthesis towards numerous biomedical applications, such as drug delivery, regenerative medicine and tissue engineering [38,39,40,41]. In general, biomedical applications of supermacroporous composite cryogels involve the chemical interaction of a ligand with the polymeric backbone. The ligand is specifically selected and designed for interaction with a biomolecule. The supermacroporous composite cryogels are commonly modified with a ligand to confirm selective binding of biomolecules. Anchoring biomolecules in scaffolds for tissue engineering protects proliferation and attachment of cells, as well as catalytically active enzymes in biomedical applications [42,43]. 

### 3.1. Purification and Separation Applications

Cryogelation has been used to produce stable and non-degradable polymeric matrices for purification and separation applications of several biomolecules. Purification and separation of target molecules from a complex medium, such as blood, urine, or cell rupture supernatant, is an important element of modern process biotechnology. Recent developments have resulted in new technologies for improved purification and separation. Traditional methods used for biomolecules purification and separation, such as ultracentrifugation and filtration, are time-consuming, expensive and very often inefficient. Moreover, using composite cryogels represent a novel approach for effective and rapid separation of biomolecules in biomedical applications [44,45,46].

**Figure 3:**
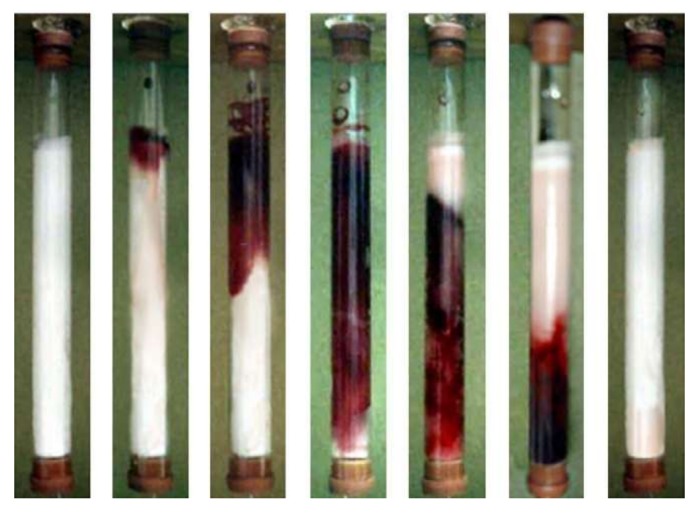
The flow pattern of human blood through a supermacroporous cryogel. Republished with permission from [47]. Copyright 2009 Elsevier.

For instance, Alkan et al. purified immunoglobulin G from human plasma with protein A-based supermacroporous cryogels. They prepared the cryogel by bulk polymerization and attached protein A to the cryogel using cyanogen bromide activation and achieved the highest adsorption capacity of 83.2 mg/g. In addition, they observed that supermacroporous structure makes it feasible to leak blood cells without blocking cryogel (Figure 3). They also obtained a high adsorption capacity from human plasma (up to 88.1 mg/g) and reused it ten times without performance loss in adsorption capacity [47].

**Figure 4:**
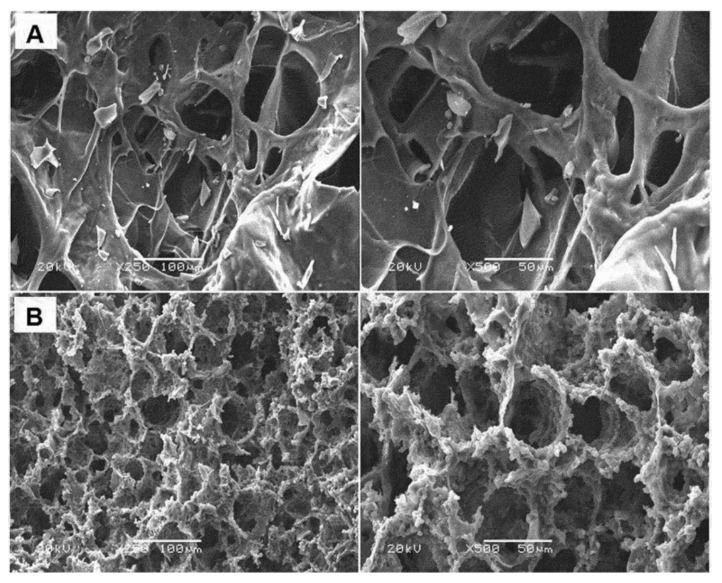
Scanning electron microscopy images of bare poly(2-hydroxyethyl methacrylate) (**A**) and embedded composite (**B**) cryogels. Republished with permission from [48]. Copyright 2014 Taylor & Francis.

Saylan et al. synthesized composite cryogels for immunoglobulin G recognition and separation based on boronate affinity chromatography. They prepared a monolith using 4-vinyl phenylboronic acid and 2-hydroxyethyl methacrylate and crushed carefully to obtain homogeneous particles. Then, the crushed particles were embedded into cryogels to prepare composite cryogels. They first characterized the composite cryogels by several experiments, such as scanning electron microscopy (Figure 4), and then investigated the separation performance of composite cryogels by high-pressure liquid chromatography system with different parameters. They also performed immunoglobulin G separation from plasma samples. The results showed that composite cryogels have flow, pressure, and other discriminative properties as an alternative material to commercial columns [48].

**Figure 5:**
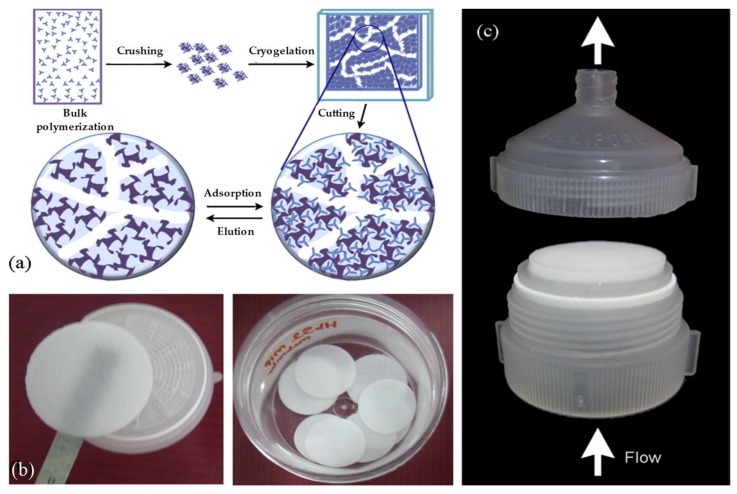
Scheme of the steps of composite cryogel formation (**a**), images of circular composite cryogels (**b**) and continuous operation setup for adsorption experiments (**c**). Republished with permission from [49]. Copyright 2012 Elsevier.

Aslıyüce et al. developed the preparation of composite cryogels for purification of hepatitis B surface antibody. They first synthesized using bulk polymerization of 2-hydroxyethyl methacrylate and hepatitis B surface antibody and then characterized hepatitis B surface antibody imprinted particles and embedded these particles onto the poly(2-hydroxyethyl methacrylate)-based cryogels (Figure 5). After the characterization experiments of composite cryogels, they performed adsorption capacity experiments and found the maximum amount was 701.4 mIU/g. They also carried out selectivity and reusability analyses of reported composite cryogel. They investigated real-sample analysis of composite cryogels and showed that it can be used for specific purification of hepatitis B surface antibody from hepatitis B surface antibody positive human plasma [49].

Bereli et al. prepared supermacroporous cryogels to purify lysozyme from egg white. They prepared l-histidine-imprinted poly(2-hydroxyethyl methacrylate)-based supermacroporous cryogels by using metal chelate monomer (*N*-methacryloyl-(l)-histidine methyl ester) due to the affinity of imidazole nitrogen donor atoms that copolymerized with 2-hydroxyethyl methacrylate monomer. After the characterization steps, they calculated the maximum lysozyme adsorption capacity as 54.2 mg/g. In addition, they performed fast protein liquid chromatography analysis to show selectivity of cryogel and demonstrated that it was able to discriminate the other structural analog ribonuclease A. The supermacroporous cryogel also possessed high storage stability and could be used several times without a decrease in adsorption capacity [50]. 

Baydemir et al. also developed composite cryogel for lysozyme purification from egg white. They prepared poly(2-hydroxyethyl methacrylate-*N*-methacryloyl-l-phenylalanine)-based particles by suspension polymerization and then embedded into a cryogel. After various characterization analyses, they performed optimization experiments with different parameters, such as pH, lysozyme concentration, temperature, flow rate, and ionic strength. They observed that the composite cryogel could be reused up to ten times in reusability studies without any significant capacity loss. Finally, the composite cryogel was used to purify lysozyme from chicken egg white, and gel electrophoresis was used to estimate the purity of lysozyme [51]. 

**Figure 6:**
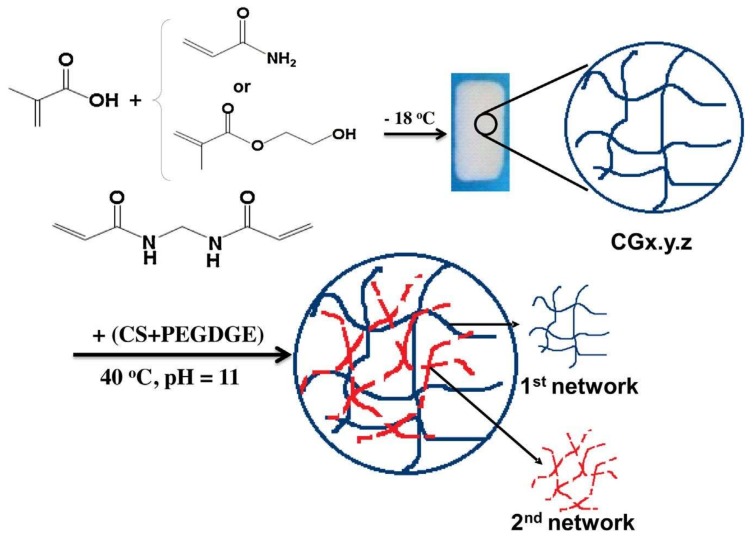
Synthesis strategy of supermacroporous composite cryogel. (CGx.y.z: first network, x: monomer ratio, y: cross-linking ratio, and z: initial monomer concentrations). Republished with permission from [52]. Copyright 2016 Elsevier.

Dragan et al. prepared supermacroporous composite cryogels, which consisted of two networks, such as methacrylic acid and either acrylamide or 2-hydroxyethyl methacrylate with chitosan as cross-linked and poly(ethylene glycol) diglycidyl (Figure 6) to load and release lysozyme. They observed that while the amount of lysozyme loaded on single network cryogel was higher than that loaded on the interpenetrating polymer network. The second network led to pore size decrease by interpenetrating polymer network, but in the presence of chitosan promoted lysozyme release from interpenetrating polymer network [52].

**Figure 7:**
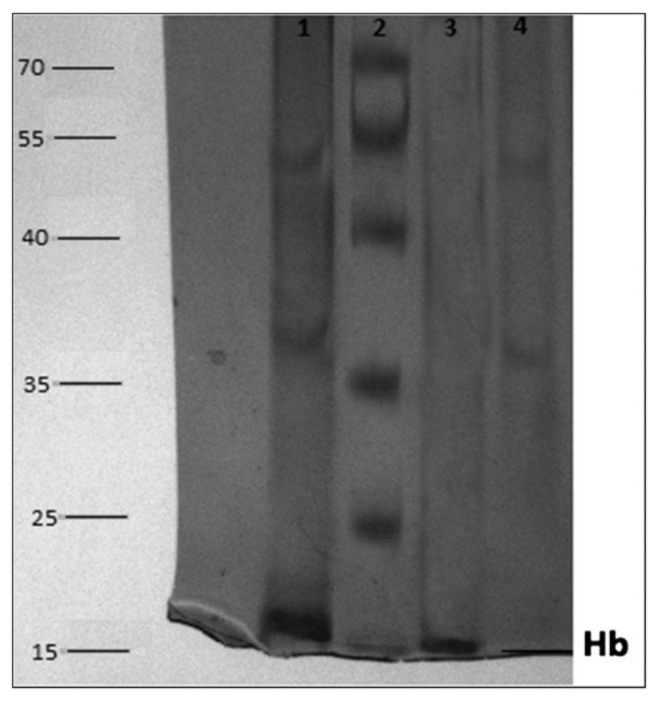
Electrophoresis image of hemolysate fractions under reducing conditions. Republished with permission from [53]; Copyright 2014 Wiley.

Baydemir et al. prepared a composite cryogel that consisted of hemoglobin imprinted poly(2-hydroxyethyl methacrylate-*N*-methacryloyl-l-histidine) particles and 2-hydroxyethyl methacrylate cryogels with high gel fraction yield up to 90% for depletion of hemoglobin. Following the characterization experiments, they showed that the composite cryogel had a high binding capacity for hemoglobin in the presence of structural analogs. They applied this in fast protein liquid chromatography for hemoglobin depletion from human blood and obtained a high depletion ratio (93.2%) by using composite cryogel. After depleted hemoglobin, they used electrophoresis system to show purified hemoglobin (Figure 7). Lanes 1, 2, 3 and 4 depict hemolysate before adsorption, protein ladder, desorbed hemoglobin and hemolysate after adsorption [53].

**Figure 8:**
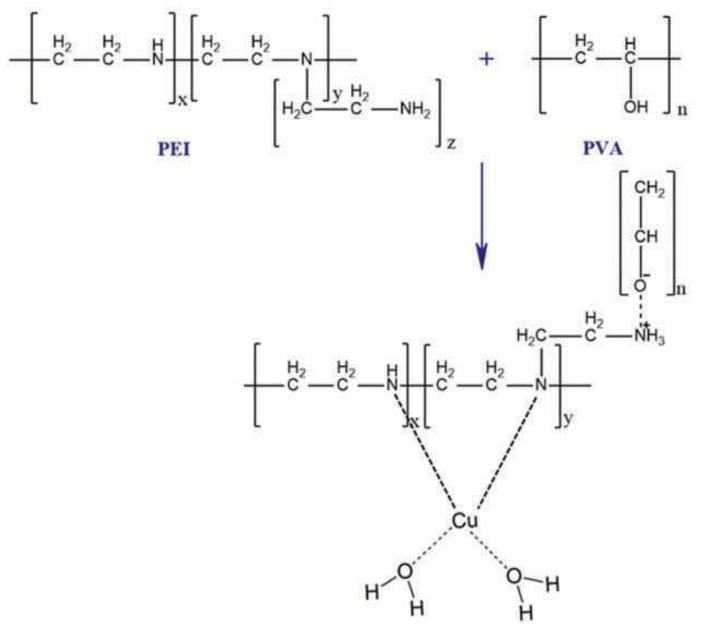
Copper chelation onto PVA/PEI composite cryogel. Republished with permission from [54]. Copyright 2016 Taylor & Francis.

Göktürk et al. provided a composite cryogel with chelated copper ions to bind and deplete hemoglobin from human blood. They first carried out the cryogelation and complexation process of polyvinyl alcohol (PVA) and polyethylene imine (PEI) with copper ion (Figure 8) and then used copper ions chelated composite cryogels for adsorption studies of hemoglobin in both aqueous solution and human plasma with different conditions, such as pH, hemoglobin concentration, adsorption time, temperature, and ionic strength. The amount of chelated copper ion was also measured by calculating initial and final concentrations of copper ions by atomic adsorption spectroscopy [54].

Baydemir et al. produced supermacroporous cryogel with embedded bilirubin-imprinted particles for the bilirubin removal from human plasma. They first synthesized poly(2-hydroxyethyl methacrylate-*N*-methacryloly-(l)-tyrosine methyl-ester)-based bilirubin-imprinted particles and then embedded these particles onto the poly(2-hydroxyethyl methacrylate)-based supermacroporous cryogel to obtain a composite polymer. They compared the adsorption capacities of non-embedded (0.2 mg/g polymer) and embedded cryogels (10.3 mg/g polymer). They also calculated the relative selectivity coefficients of composite cryogel for bilirubin/cholesterol and bilirubin/testosterone and found that they were 8.6 and 4.1 times greater than non-embedded cryogel, respectively. They utilized composite cryogel many times without decreasing adsorption capacity [55]. 

Perçin et al. developed a poly(2-hydroxyethyl methacrylate)-based cryogel disc that consisted of *N*-methacryloyl-l-tryptophan methyl ester monomer for removal of bilirubin from human plasma. They produced the cryogel disc with high gelation yield (92%) and characterized by several analyses including swelling tests, elemental, scanning electron microscopy, contact angle, Brunauer–Emmett–Teller, and surface energy calculations. They performed bilirubin adsorption studies in a batch system and found the maximum bilirubin adsorption capacity was 22.2 mg/g on cryogel discs. They also examined reusability performance and concluded it was considerably high. Thus, these polymers provide economic advantages and good properties as affinity discs for removal of bilirubin from human plasma [56]. 

Li et al. fabricated supermacroporous composite cryogels based on graphene oxide by a chemical reduction method. At first, chitosan was introduced in cryogels during the reduction process to improve their mechanical properties. Then, they characterized the composite cryogels reinforced with chitosan by several measurements and next, they examined the adsorption capacity of cryogels for bilirubin. The results showed that the composite cryogel had a high adsorption capacity (458.9 mg/g) and a fast adsorption rate. In addition, the composite cryogel also had a low hemolysis ratio and negligible anti-coagulant activity that could suggest good compatibility and high capacity adsorption, making it a potential candidate for blood samples [57]. 

Kavoshchian et al. also prepared human serum albumin immobilized poly(2-hydroxyethyl methacrylate) composite cryogel as an alternative sorbent in hemoperfusion columns for bilirubin removal from serum. They used cyanogen bromide as a matrix-activating agent for the preparation of immobilized composite cryogels. They characterized the composite cryogels by various analyses and then performed the removal of bilirubin from plasma samples. They analyzed several parameters that affect the adsorption capacity of composite cryogel, including incubation time, albumin concentration, bilirubin concentration in plasma, and temperature, and found that the maximum bilirubin removal from plasma was 25.4 mg/g at 37.5 °C [58]. 

La Spina et al. developed a poly(2-hydroxyethyl methacrylate)-based composite cryogel functionalized with l-lysine and characterized for heparin adsorption. They examined the maximum adsorption capacity using aqueous (40,500 IU heparin/g cryogel) and serum sample solutions (32,500 IU heparin/g cryogel). Therefore, they also evaluated heparin adsorption (4330 IU/g cryogel, 87% of the initial concentration) in human plasma spiked with 100 IU/mL of heparin. Under the same conditions, the non-specific adsorption of heparin onto the bare poly(2-hydroxyethyl methacrylate) cryogel was 8% of the initial amount. They reported that the composite cryogels showed good blood compatibility, as indicated by negligible adsorption of albumin, anti-thrombin III, and total protein, thus, it is suitable for extracorporeal heparin removal [59].

Tijink et al. developed cryogel membranes using the material SlipSkin in different ratios, which is a copolymer, made from *N*-vinylpyrrolidone and butyl methacrylate for blood purification. They focused on understanding the mechanism of pore formation and tailoring of cryogel membrane properties for blood purification therapies and observed that the polymer composition, solvent type and solvent evaporation time influence the cryogel membrane morphology. They claimed that the cryogel membranes had good blood compatibility properties as they have lower platelet adhesion in contact activation, thrombogenicity, leukocyte adhesion, hemolysis, and complement activation and were also very good and comparable to the benchmarks [60].

**Figure 9:**
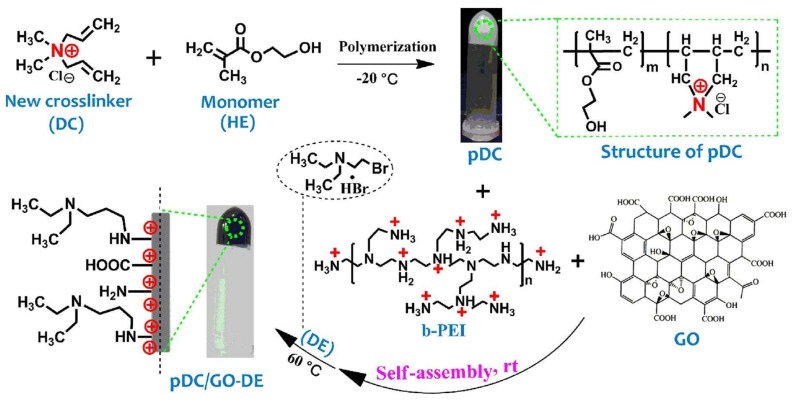
Scheme of composite cryogels preparation. Republished with permission from [61]. Copyright 2019 Elsevier.

Zhao et al. evaluated a new composite cryogel containing diallyl dimethyl ammonium chloride and 2-hydroxyethyl methacrylate that was modified with graphene oxide and *N*-diethylethanamine hydrobromide to purify several proteins and their mixtures (Figure 9). Furthermore, the supermacroporous structure supported size-selective purification. They diluted human serum ten times and observed that almost 100% of proteins were adsorbed in a short time, and the interference of the matrix was minimized. They also obtained a high extraction ratio of proteins in human serum repeatedly for three usages of cryogel [61].

### 3.2. Tissue Engineering Applications

Cryogel scaffolds have recently become popular as a template for regeneration of various tissues. There is growing literature that demonstrates their applicability in tissue engineering with extensive information provided regarding fabrication, porosity, and mechanical integrity of the scaffolds. The typical characterizations of composite cryogels involve analysis of overall interconnectivity for ensuring appropriate properties for a specific tissue. The mechanical durability can also be assessed through ultimate compression, as well as the application of multiple loads over an extended period of time. All these advantages mean that composite cryogels can advance the technology and expand tissue engineering research in biomedical applications [62,63,64].

Biçen Ünlüer et al. synthesized composite cryogels for separation and purification of hyaluronic acid. Firstly, they synthesized d-glucuronic acid-imprinted particles by using metal chelate monomer (*N*-methacryloyl-l-histidine methyl ester) and then embedded onto the acrylamide-based cryogels to obtain supermacroporous composite cryogels. After that, they characterized the composite cryogels by several methods and then performed adsorption experiment to obtain the maximum adsorption capacity (318 mg/g). They investigated the selectivity and recovery performance of composite cryogels and reported that these cryogels could be reused without performance loss. In addition, they combined the selectivity and mechanical properties of composite cryogel and obtained a rigid and stable material [65].

**Figure 10:**
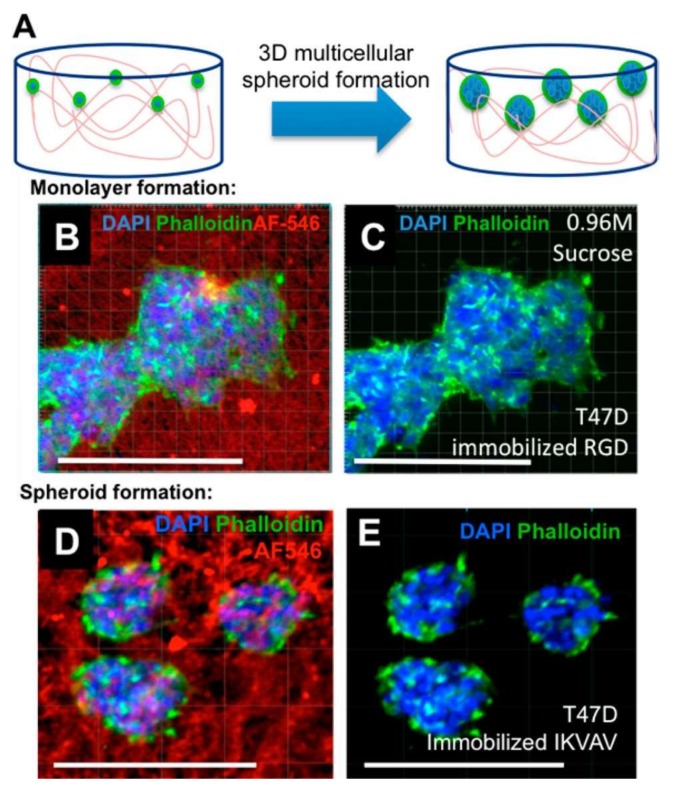
Scheme of cells cultured on cryogels (**A**), fluorescently labeled cryogels immobilized with peptides result in cell monolayers (**B**,**C**) and cellular spheroids (**D**,**E**). Republished with permission from [66]. Copyright 2016 ACS.

Tam et al. also synthesized biomimetic cryogels and gained insights into the mechanism of their formation. They examined the mono/disaccharide additives effect on the size of pores and how they interact with polysaccharide polymers to alter cryogel pore size and mechanical properties. In addition, they demonstrated both optical transparency with three-dimensional spatial control of immobilized bioactive growth factors using multiphoton patterning and cellular response to immobilized ligands. T47D breast cancer cells cultured in cryogels are depicted in Figure 10. The blue color are nuclei (DAPI), green is actin (phalloidin−AlexaFluor 488) and red cryogel pore walls represent (HA−furan−AlexaFluor 546) [66].

Petrov et al. fabricated electrically conducting 2-hydroxyethyl cellulose/polyaniline-based composite cryogels by combining cryogenic treatment and photochemical cross-linkers. They first placed polyaniline nanofillers synthesized via oxidative polymerization of aniline in aqueous media and, then embedded them in the 2-hydroxyethyl cellulose matrix. They studied the effect of polyaniline amount morphology on the cryogel fraction yield, electrical conductivity of the material and found high cryogel fraction yield was from 65 to 95%. They also carried out a cytotoxicity test on composite cryogels and the results demonstrated high survival and proliferation of cells on the composite cryogels in the presence of the electrical field [67]. 

Von der Ehe et al. synthesized a supermacroporous cryogel and functionalized with an atom transfer radical polymerization initiator at the surface. They introduced two new glycol monomers which possess deprotected mannose as well as glucose moieties. They used *N*-isopropylacrylamide to copolymerize the cryogel surface and provided a highly hydrophilic supermacroporous cryogel. After the characterization analyses, they exemplified by investigating the ability to capture *Escherichia coli* and revealing binding interactions of the bacteria with the mannose glycopolymer-functionalized cryogel. According to the results, they reported that the composite cryogel represented a promising material for affinity chromatography and enrichment of cells [68]. 

Erdem et al. developed cryogels from difunctional Jeffamine and glutaraldehyde in the presence of a trifunctional Jeffamine as a cross-linker. These cryogels demonstrated high pore interconnectivity and functionality and also permitted cell adherence and growth while maintaining mechanical strength, biodegradability, and biocompatibility without causing any immune response. They investigated the visco-elastic behavior of the cryogels by unconfined compression test which depicted good elastic modulus and mechanical strength. Furthermore, they evaluated in vitro biocompatibility by cytotoxicity test using human umbilical vein endothelial cells for cell growth on the non-reduced and reduced Jeffamine cryogels [69]. 

**Figure 11:**
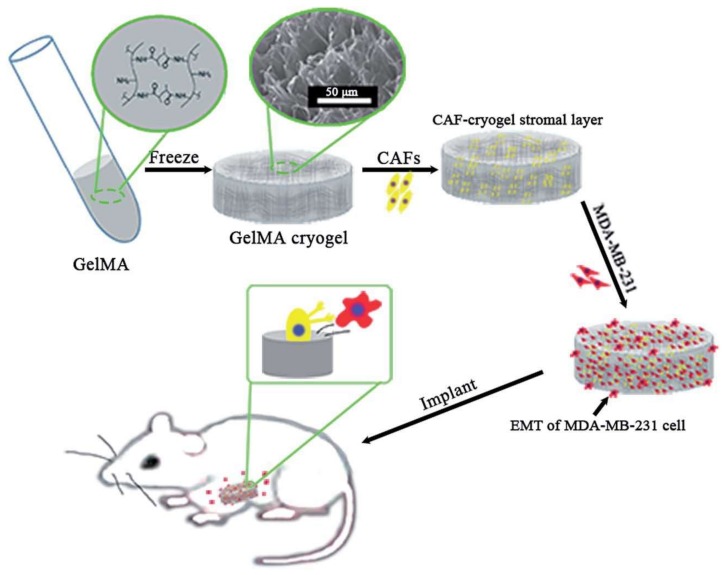
The co-culture system for the role of carcinoma-associated fibroblasts in the epithelial–mesenchymal transition process of human breast cancer cells in vitro and in vivo. Republished with permission from [70]. Copyright 2017 the Royal Society of Chemistry.

Zhang et al. exhibited gelatin and methacrylic anhydride (GelMA)-based three-dimensional cryogel co-culture system to evaluate the role of carcinoma-associated fibroblasts in breast cancer metastasis (Figure 11). They found that carcinoma-associated fibroblasts (CAFs) could promote the epithelial–mesenchymal transition of human breast cancer (MDA-MB-231) cells in a three-dimensional cryogel co-culture system in vitro. After that, they used the co-culture human breast cancer system to construct a xenograft tumor mouse model for in vivo studies. The results showed that carcinoma-associated fibroblasts could also promote deterioration and metastasis of human breast cancer cells in a xenograft tumor [70].

Konovalova et al. reported the preparation of composite cryogels using pectin and chitosan. They found the structural and physicochemical properties of composite cryogels changed with pectin and chitosan amount. They observed that the addition of chitosan to composite cryogels can increase their mechanical strength, cause the change in surface morphology, increase the degradation time and enhance adhesion. Apple pectin-based cryogels were observed to be less immunogenic as compared with *Heracleum L*. pectin based cryogels. In addition, apple pectin- and *Heracleum L*. pectin-based cryogels were hemocompatible cryogels and the percentage of red blood cells hemolysis was less than 5% [71]. 

**Figure 12:**
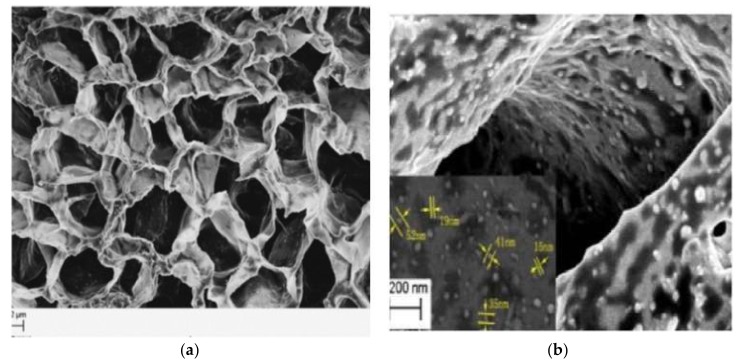
Scanning electron microscopy images of (**a**) cryogel and (**b**) composite cryogel. Republished with permission from [72]. Copyright 2017 Taylor & Francis.

Zou et al. synthesized cryogels based on methacrylated carboxymethyl chitosan and poly(ethylene glycol) diacrylate precursors. Due to its excellent properties, such as fast swelling behavior, inter-connective porous structure, high water absorption capacity, and especially the presence of abundant carboxyl methyl groups on its backbone. The cryogels not only favored the absorption of silver ions but also proved to be a good matrix for the incorporation of silver nanoparticles by in situ chemical reduction (Figure 12). They performed an inhibition zone test and anti-bacterial inhibition ratios, which indicated the composite cryogel had prominent and durable anti-bacterial activity against Gram-negative *E. coli* and could be utilized as a potential anti-bacterial material [72].

**Figure 13:**
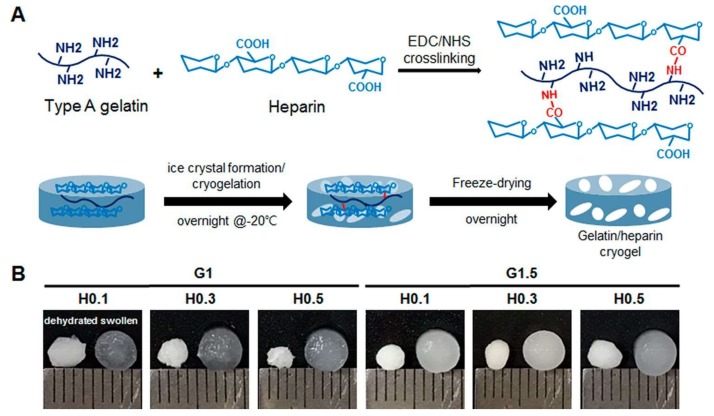
Preparation of composite cryogel (**A**) and mechanical properties in different gelatin concentration (**B**). Republished with permission from [73]. Copyright 2018 ACS.

Kim et al. developed an injectable cryogel by conjugating heparin into gelatin as a carrier for vascular endothelial growth factor and fibroblasts in hindlimb ischemic disease. Their composite cryogel showed gelatin concentration-dependent mechanical properties, swelling ratio, interconnected porosity, and elasticity. As seen Figure 13, gelatin was cross-linked with heparin under EDC/NHS mediated cryogelation process and gelatin/heparin-based cryogels fabricated with the combination of 1, 1.5% gelatin (G1, G1.5) and 0.1, 0.3, 0.5% heparin (H0.1, H0.3, H0.5). Furthermore, the controlled release of vascular endothelial growth factor led to effective angiogenic responses both in vitro and in vivo applications. They also showed that composite cryogels’ sponge-like properties enabled them to be applied as an injectable carrier system for in vivo cells and growth factor delivery [73].

Sedlacık et al. reported a recent study about the potential of poly[*N*^5^-(2-hydroxyethyl)-l-glutamine-stat-*N*^5^-(2-methacryloyl-oxyethyl)-l-glutamine] with 2-hydroxyethyl methacrylate and *N*-propargyl methacrylamide-based supermacroporous cryogels as biodegradable polymers to support proliferation and chondrogenesis of human dental pulp stem cells. They prepared two types of cryogels with different compressive moduli and modified each type with two different concentrations of cell supporting moieties (peptides). They used X-ray computed nanotomography to visualize and analyze the three-dimensional structure of the cryogels, and results showed that modifying cryogels within the range of peptide concentrations had a positive effect on the proliferation of human dental pulp stem cells [74]. 

### 3.3. Drug Delivery Applications

The composite cryogels have great potential for controlled drug release and stimulus-responsive behavior. In drug delivery systems, the polymer chain has more widely controllable physical properties and also more efficient drug loading/release properties have been induced by the introduction of additional comonomers. The composite cryrogels are widely studied as vehicles for drug delivery. Sustained and localized drug delivery can enhance the safety and activity of drugs in diverse disease settings in biomedical applications [75,76,77]. 

Bakhshpour et al. prepared anti-biotic and anti-neoplastic chemotherapy drug (mitomycin C) imprinted cryogel membranes for mitomycin C recognition. They synthesized different ratios of 2-hydroxyethyl methacrylate and methylene bisacrylamide-based membranes by using metal ion coordination interactions with *N*-methacryloyl-(l)-histidine methyl ester and characterized the cryogel membranes by several analyses and investigated cytotoxicity of cryogel membranes using mouse fibroblast cell line L929. They performed time-dependent release and cryogel membranes demonstrated very high mitomycin C loading efficiency (70–80%) and sustained mitomycin C release over hours [78]. 

Öncel et al. also designed poly(2-hydroxyethyl methacrylate-*N*-methacryloyl-l-glutamic acid)-based cryogel for the delivery of mitomycin C. They carried out in vitro drug delivery studies to examine the effects of cross-linker ratio and template amount and observed that the cumulative release of mitomycin C was decreased with an increase in cross-linking agent ratio and higher template amount in cryogel structure. Cytotoxicity behavior of cryogel was investigated using mouse fibroblast cell line L929, and the results showed that L929 cell viability of cryogel was measured as 97.27 ± 3.57, which means it is not toxic [79].

**Figure 14:**
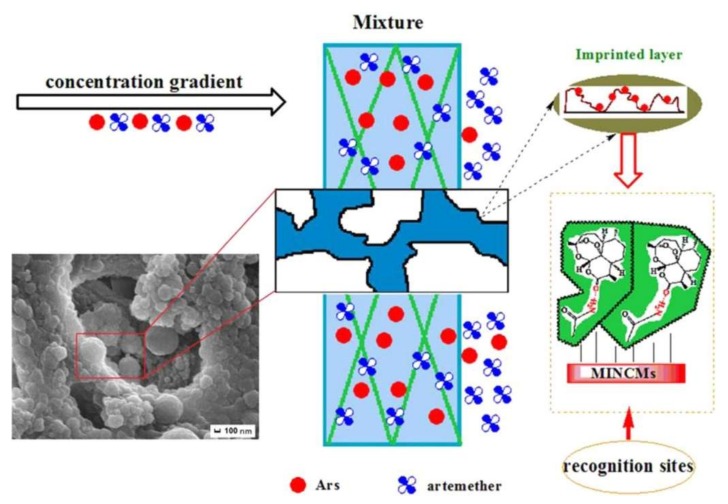
Separation mechanism for artemisinin and artemether toward composite cryogel membranes. Republished with permission from [80]. Copyright 2015 Elsevier.

Wu et al. developed composite cryogel membranes for artemisinin recognition. They first prepared a hybrid structure at the surface of polydopamine-modified cellulose membranes and then synthesized composite membranes (Figure 14). Furthermore, the composite membranes did not only show a high adsorption capacity (54 mg/g) but also had an excellent perm-selectivity with high separation factor (15.03) towards artemisinin. Reported material shows encouraging behavior for separation and purification of artemisinin [80]. Wu et al. also prepared thermally responsive composite cryogel membranes for efficient selective adsorption and separation performance toward ciprofloxacin. Due to the formation of the polydopamine coating surfaces, they obtained outstandingly enhanced rebinding capacity (2.343 mmol/cm^2^). They reported that thermally responsive composite membranes demonstrated a high temperature-dependent switching mode for separation of the ciprofloxacin and had a high adsorption regeneration performance (92.74%). They finally claimed that this composite membrane preparation strategy can be used in a wide range of biomedical applications [81].

**Figure 15:**
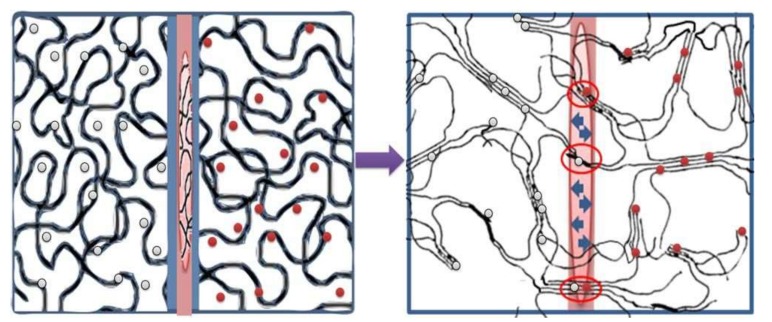
Scheme of bilayer synthesis cryogels. Republished with permission from [82]. Copyright 2018 Wiley.

Lima et al. presented cryogels synthesis with drugs ceramics by physical cross-linker for theophylline release and stimulus-responsive behavior. They produced a layer-by-layer structure by adding both cryogels ceramics into a mold (Figure 15). The cryogels were produced from polyvinyl alcohol and polyacrylic acid by varying the molecular weight of the polymers and they showed a barrier of integration with physical cross-linking. Swelling of composite cryogels showed drug release from both cryogels ceramics with simultaneous, independent drug release without diffusing by opposite layer. In addition, the ceramic can enhance the mechanical properties of composite within the cryogel and can be tailored for specific drugs and drug release profiles [82]. 

### 3.4. Other Applications

Composite cryogels are the key class of polymeric materials and are also widely used in the biomedical applications as drug delivery systems, matrices for cell immobilization, scaffolds for tissue engineering, biomaterials, biosensors and wound dressing. These materials offer new perspectives for the development of innovative systems in biomedical applications. Different types of composite cryogels in varying physical forms have been used for adsorption of small-molecule drugs, proteins, oligonucleotides, silencing RNA, plasmid DNA, and antibodies [83,84,85].

Ergün et al. prepared Fe^3+^ imprinted beads embedded composite cryogel for removal of Fe^3+^ ions from the plasma of a β-thalassemia patient. They first synthesized *N*-methacryloyl-(l)-cysteine methyl ester-based beads that coordinated with Fe^3+^ ions and then embedded into glycidyl methacrylate based cryogel matrix. They measured the surface area, swelling degree and maximum adsorption capacity of composite cryogel as 76.8 m^2^/g, 7.7 g H_2_O/g cryogel and 2.23 mg/g. They also calculated the relative selectivity of composite cryogel towards Fe^3+^ ions and found that it was 135.0, 61.4, and 57.0 times greater than Ni^2+^, Zn^2+^, and Fe^2+^ ions, respectively [86]. 

Berezhna et al. developed supermacroporous composite cryogel with embedded divinylbenzene-styrene particles using polyvinyl alcohol or agarose solutions. They characterized the scanning electron microscopy analyses to show multiple interconnected pores and quite homogeneous distribution of particles in the reported composite cryogels. They assessed the biocompatibility of composite cryogels by estimation of the C5a fragment of complement in the serum sample and concentration of fibrinogen in the blood plasma which contacted the composite cryogels. A time-dependent generation of C5a fragment indicated weak activation of the complement system [87]. 

Akande et al. synthesized poly(2-hydroxyethyl methacrylate) based composite cryogels at −12 °C for 18 h and produced spongy, elastic and macroporous polymers that were highly reproducible with over 90% recovery. They tested the mechanical properties of composite cryogel, such as storage modulus, compressive modulus, and creep test, with the dynamic mechanical analyzer. The results showed that the composite cryogel matrix had high recovery after 70% compression of cryogel and a compressive modulus of 1.8 to 8.5 kPa [88]. 

Savina et al. proposed a new method for the synthesis of supermacroporous composite cryogels. They synthesized by controlling the freezing step of the monomer solution, three-dimensional supermacroporous cryogels with wide interconnected pores and large accessible surface area were produced. They also manufactured supermacroporous acrylamide-based composite cryogels with potential for regulating much larger cryogel dimensions. They claimed that this method could be employed for multi-component supermacroporous composite materials production with homogenously embedded particles and serve as a biomaterial in biomedical, biotechnological and environmental applications [89]. 

**Figure 16:**
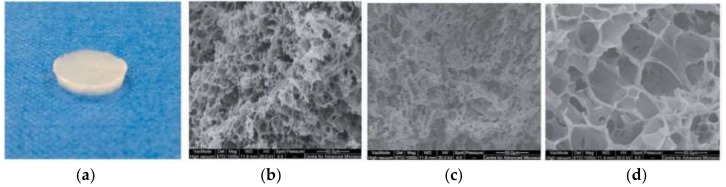
Optic image of swollen cryogel with 0.8% NaOH (**a**) and scanning electron microscopy images of poly(vinyl alcohol)–Gantrez® AN based cryogels in the presence of 0.4% (**b**), 0.6% (**c**) and 0.8% NaOH (**d**). Republished with permission from [90]. Copyright 2016 the Royal Society of Chemistry.

Calo et al. produced polyvinyl alcohol-based supermacroporous cryogels from the aqueous mixtures with different amount of NaOH, which were frozen at −18 °C for 9 h and then thawed at 25 °C (Figure 16). They reported that these cryogels exhibited superior mechanical properties and high swelling ability in water. They also observed that these supermacroporous cryogels exhibited intrinsic anti-microbial properties against *Staphylococcus aureus* without any anti-microbial agent. They reported that in vitro biocompatibility of supermacroporous cryogels was assessed using human dermal fibroblasts with very encouraging results [90].

## 4. Conclusions and Outlooks

During the past several decades, supermacroporous composite cryogels have already utilized a dramatic effect in biomedical applications. Their application for biomolecules purification, separation and recognition have accoladed. A number of well-documented studies have demonstrated the physical, chemical and mechanical properties of cryogels are pivotal and distinctive. They act as tissue scaffolds, membranes, drug delivery agents, adsorbents, and bioreactors with many preparation methods, which can provide all the necessary information. In the area of soft materials processing and refinement, there is a still plenty of room for advancement.

Herein, a variety of supermacroporous composite cryogels were reviewed specifically for biomedical applications. The reported results show that the interaction types between monomers and templates significantly change the rebinding features of biomolecules to supermacroporous composite cryogels. Furthermore, the supermacroporous composite cryogels have a high surface area and binding capacity by embedding particles. The recent studies are promising for further development of functionalized supermacroporous composite cryogels that can be applied to purify, recognize, and separate a variety of biomolecules.
